# The involvement of family in the Dutch practice of euthanasia and physician assisted suicide: a systematic mixed studies review

**DOI:** 10.1186/s12910-019-0361-2

**Published:** 2019-04-05

**Authors:** Bernadette Roest, Margo Trappenburg, Carlo Leget

**Affiliations:** University of Humanistic Studies, Kromme Nieuwegracht 29, 3512HD Utrecht, The Netherlands

**Keywords:** Physician-assisted dying, Euthanasia, Family, End-of-life, Decision-making

## Abstract

**Background:**

Family members do not have an official position in the practice of euthanasia and physician assisted suicide (EAS) in the Netherlands according to statutory regulations and related guidelines. However, recent empirical findings on the influence of family members on EAS decision-making raise practical and ethical questions. Therefore, the aim of this review is to explore how family members are involved in the Dutch practice of EAS according to empirical research, and to map out themes that could serve as a starting point for further empirical and ethical inquiry.

**Methods:**

A systematic mixed studies review was performed. The databases Pubmed, Embase, PsycInfo, and Emcare were searched to identify empirical studies describing any aspect of the involvement of family members before, during and after EAS in the Netherlands from 1980 till 2018. Thematic analysis was chosen as method to synthesize the quantitative and qualitative studies.

**Results:**

Sixty-six studies were identified. Only 14 studies had family members themselves as study participants. Four themes emerged from the thematic analysis. 1) Family-related reasons (not) to request EAS. 2) Roles and responsibilities of family members during EAS decision-making and performance. 3) Families’ experiences and grief after EAS. 4) Family and ‘the good euthanasia death’ according to Dutch physicians.

**Conclusion:**

Family members seem to be active participants in EAS decision-making, which goes hand in hand with ambivalent feelings and experiences. Considerations about family members and the social context appear to be very important for patients and physicians when they request or grant a request for EAS. Although further empirical research is needed to assess the depth and generalizability of the results, this review provides a new perspective on EAS decision-making and challenges the Dutch ethical-legal framework of EAS. Euthanasia decision-making is typically framed in the patient-physician dyad, while a patient-physician-family triad seems more appropriate to describe what happens in clinical practice. This perspective raises questions about the interpretation of autonomy, the origins of suffering underlying requests for EAS, and the responsibilities of physicians during EAS decision-making.

## Background

Euthanasia and physician assisted suicide (EAS) seems to be an accepted practice in the Netherlands, although the legislation and practical implications of EAS are still subject to intense debate [1, 2]. Both in 1992 and in 2017, public acceptance of EAS was found to be around 90% [3]. In the Netherlands, euthanasia is defined as the active termination of a patient’s life at their explicit request, by a physician who administers a lethal substance to the patient [4]. In physician assisted suicide, a physician supplies the lethal substance to a patient who ingests the substance in the presence of the physician.

Dutch physicians are not persecuted for performing EAS if they comply with the due care criteria as formulated in the euthanasia law. First, the physician must be convinced that the patient’s request for EAS is well-considered and voluntary, and that the patient’s suffering is lasting and unbearable. In addition, the patient has to be informed about his situation and prognosis, there must be no other reasonable solution and a second, independent physician has to be consulted. Last, the termination of a life or an assisted suicide has to be performed with due care [4]. After EAS has been performed, the physician must notify a municipal pathologist and reports written by the physician and the independent consultant are sent to a regional euthanasia review committee that evaluates whether the due care criteria have been met.

While the Dutch euthanasia law was enacted in 2002, the performance of and empirical research on EAS already started in the 1980s [5, 6]. Regularly performed empirical studies show that EAS is still relatively rare. In 2015, euthanasia accounted for 4.5% of annual deaths, physician assisted suicide for only 0.1% [7]. According to the latest annual report of the Dutch euthanasia review committees, EAS was carried out most often by general practitioners (GPs), namely in 85% of cases. In 80% of all cases EAS took place at home and in 65% of the cases it involved patients with incurable cancer [8].

The family’s role in the Dutch practice of euthanasia and assisted suicide has been receiving critical attention lately, although their involvement had already been documented before and shortly after the enactment of the euthanasia law [9, 10]. Recent qualitative studies describe how family members such as partners and children can influence the process of euthanasia decision-making and how some physicians take family members’ well-being and bereavement into account when deciding whether or not to grant a request [11, 12]. In contrast to these findings, the Dutch euthanasia law does not consider the position of family members at all, except that requests for EAS need to be free of undue pressure. Dutch clinical guidelines on EAS also barely describe the position and relevance of family members in EAS decision-making [13]. Hence, empirical findings on the involvement of family members in the practice of EAS raise practical and ethical questions, which require further examination from both an empirical and ethical perspective [11, 14, 15].

To date, a systematic review of empirical research addressing the involvement of family members in the Dutch practice of EAS has not been performed. Several authors have described different aspects of family involvement, such as the different roles family members may take in euthanasia decision-making [10], the bereavement process of relatives after EAS [14, 16] and the potential influence of family members’ suffering on end-of-life decision-making [15]. However, there is no comprehensive overview that incorporates all elements that might be relevant for the Dutch practice of EAS. Meanwhile, there is a growing body of literature in the fields of medical ethics and palliative care that underlines the relevance of the patient’s significant others in medical decision-making and its consequences for clinical practice, and several authors have called for further empirical inquiry [17–25].

Therefore, the aim of this review is to explore how family members are involved in the Dutch practice of EAS, according to empirical research, and to map out themes that could serve as a starting point for further empirical research and ethical discussion. A systematic review was performed with a broad research question: what do both qualitative and quantitative studies on EAS from the Netherlands reveal regarding the involvement of family members before, during and after EAS? The question who the ‘family members’ are is part of this research question. In the context of Dutch healthcare, the term ‘family’ is mostly used for (marital) partners and first-degree blood relatives (parents, children and siblings). However, a patient’s social network may be constituted differently, and people other than marital partners or blood relatives may be closer to the patient and may be far more important in the process of medical decision-making [26]. Therefore, this specific point needs close examination as well. Notwithstanding the focus on the Dutch situation, the results of this study could offer new insights into the practice of physician assisted dying generally, and could inform both the national and international debate on its legislation and practical implications.

## Methods

A systematic search strategy for mixed studies reviews was used, following the PRISMA guidelines [27, 28]. First, a primary search was carried out in the databases Pubmed, Embase, PsycInfo, and Emcare with the use of the search strategy as displayed in Table 1. Second, some additional articles were retrieved by snowballing and checking references. Additionally, experts in the field were asked about key documents on the topic. Two researchers screened titles and abstracts of the retrieved articles to determine their eligibility for the review. This was followed by a full-text screening of the remaining articles by three researchers. The inclusion criteria were original, empirical, scholarly research considering any aspect of the involvement of family members in the Dutch euthanasia practice from 1980 until 2017, published in English or Dutch. Disagreements on the inclusion or exclusion of articles were discussed among the three researchers. If necessary, authors were contacted for additional information.

**Table 1:** Search strategy

Search #1: Euthanasia OR assisted suicide OR physician-assisted suicide OR physician-assisted dying	
Search #2: Netherlands OR Dutch OR Netherland* OR Holland	
Search #3: Family OR families OR caregivers OR relatives OR partner OR children OR friends	
Search #4: #1 AND #2 AND #3	

Early in the process of reviewing the empirical studies, it became clear that there was much variety in terms of objectives, methods and quality, and that there were differences in the underlying epistemological positions. In addition, the involvement of family members was not the primary objective of study in a considerable number of studies, though they were referred to in the results. To come to a meaningful synthesis in light of this heterogeneity, an inductive qualitative approach was chosen for the synthesis of the included studies [29]. To synthesize the results of both qualitative and quantitative studies, thematic analysis was adopted as method, which is a useful approach when describing and mapping out an underexplored area [30, 31]. Following this approach, studies were not excluded based on their quality. The first researcher performed line-by-line coding of the results sections of the included studies and attributed descriptive themes. The descriptive themes were discussed among the three researchers to reach consensus on their accuracy and meaning. Subsequently, analytical themes that overarch the descriptive themes were developed during group discussions and were tested for their soundness in the included studies*.* To enhance the researchers’ reflexivity, note-taking, group discussions on personal judgments and an active search for disconfirming cases were carried out during the review process.

## Results

**Fig. 1:**
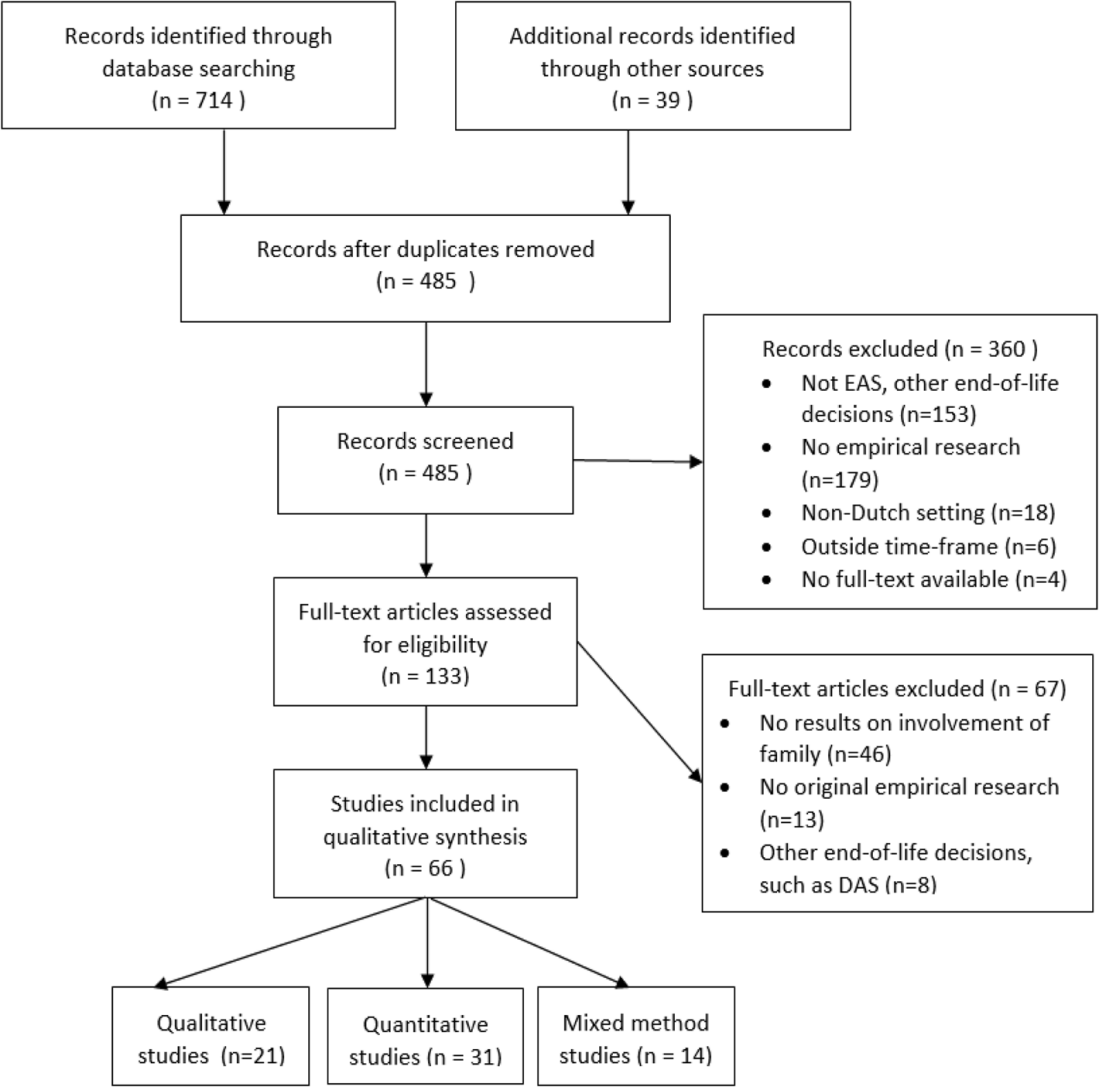
PRISMA flowchart systematic search and selection

The systematic search and selection process yielded 66 studies that met the inclusion criteria. The selection process is presented in the flow diagram in Fig. 1 and the main characteristics of the included studies are displayed in Table 2. Only 14 of the 66 included studies had family members as study participants. Some studies described family members as study participants, although their own opinions or experiences did not seem to be a topic of research, and the results made no further mention of them [32, 33]. These studies were excluded from further analysis. We also excluded a number of studies presenting results on demedicalized assistance in dying (DAS). For the ethnographic studies, the original text was included instead of articles derived from the original studies. Finally, the official governmental evaluations of the euthanasia law were included even though one might consider them grey literature, because they are an important source of empirical data.

**Table 2:** Main characteristics of the included studies

Author	Year	Objective	Study type	Participants/Setting
Qualitative Studies				
Meyboom-de Jong [36]	1983	To discuss 4 cases of active life termination on request	case study	cases of patients with cancer (n = 3) and obstructive lung disease (n = 1) who died by euthanasia, recounted by different GPs
Ponsioen [44]	1983	To discuss 3 cases of active life termination on request	case study	cases of patients with cancer (*n* = 2) and ALS (*n* = 1) who died by euthanasia reported by a GP
Koerselman [68]	1986	To describe cases of patients exercising their autonomy	case study	case of a daughter of a cancer patient who died after euthanasia (n = 1), reported by a psychiatrist
Ponsioen [65]	1997	To evaluate the role of the consulting physician in the context of EAS in the homecare setting	case study	cases of patients with cancer (n = 2) recounted by a GP
Pool [9]	1996	To describe the euthanasia practice in one Dutch hospital	ethnographic research	In-hospital observations of and interviews with patients (n = + − 50) with end-stage diseases, involved family members and attending healthcare professionals
The [34]	1997	To describe the role of nurses in euthanasia cases and other end-of-life decisions in one Dutch hospital	ethnographic research	In-hospital observations of and interviews with patients with end-stage lung disease (*n* = 50), the involved nurses, family members and other healthcare professionals
Norwood [35]	2009	To describe the day -to-day experience of EAS decision-making and performance in Dutch family practice	ethnographic research	observations of and interviews with GPs and patients (*n* = 14 and *n* = 650), case study research with a selection of GPs (*n* = 10) and their end-of-life patients (*n* = 25)
Marwijk et al. [78]	2007	To obtain further insight into the emotional impact on GPs of performing euthanasia or assisted suicide, and to tailor the educational needs of vocational GP-trainees accordingly	focus groups	GPs (*N* = 22)
Ciesielski-Carlucci and Kimsma [38]	2000	To recount the stories of physicians and families who have experience with assisted dying	in-depth interviews	physicians who ever have performed EAS (*n* = 11), relatives of patients who died by EAS (*n* = 12)
Georges et al. [81]	2008	To describe GPs’ experiences in dealing with requests for EAS from terminally ill patients and to gain insight into factors that are related to their restrictive attitude towards euthanasia	in-depth interviews	GPs (*n* = 30)
Pasman et al. [46]	2009	To examine how patients whose request for euthanasia was not granted or performed described their suffering, how their physicians assessed suffering in those cases and how they describe unbearable suffering in general.	in-depth interviews	patients with advance directives whose EAS request was refused (n = 10) and treating physicians (*n* = 16, mostly GPs)
Rurup et al. [39]	2010	To examine how physicians and patients think about ADs for euthanasia in the case of dementia, how they experience the discussion of discussing ADs for dementia, what they expect about the use of ADs in clinical practice	in-depth interviews	patients with an advanced directive due to dementia of the fear of dementia (n = 7), relatives (*n* = 4) and their physicians (*n* = 9)
Buiting et al. [76]	2011	To investigate whether and how palliative treatment alternatives come up during and before euthanasia consultations and how the availability of possible palliative treatment alternatives are assessed	in-depth interviews	consulting physicians (*n* = 14), physicians who had requested an euthanasia consultation (*n* = 12)
Dees et al. [45]	2011	To explore what patients who had explicitly requested EAS considered ‘suffering’ to entail and what made their suffering ‘unbearable’.	in-depth interviews	patients with an explicit request for EAS (*n* = 31)
Booij et al. [37]	2013	To obtain in-depth information about patients’ thoughts on and attitudes towards euthanasia, PAS and the use of advance directives in HD and to assess the difficulties patients encounter when thinking about end-of-life wishes	in-depth interviews	patients with Huntington’s disease (*n* = 14)
Dees et al. [64]	2013	To explore the decision-making process in cases where patients request euthanasia and to understand the different themes relevant to the optimization of this decision-making process	in-depth interviews	patients with an explicit request for EAS (n = 31), the most-closely involved relatives (n = 31), treating physicians (*n* = 28, mostly GPs).
Snijdewind et al. [11]	2014	To identify and categorize the characteristics of EAS requests that are more complex than others	in-depth interviews	physicians (n = 28, mainly GPs), relatives of patients whose EAS request was granted or refused (n = 22)
Kouwenhoven et al. [41]	2015	To investigate the differing opinions of physicians and members of the general public on the acceptability of euthanasia in patients with advanced dementia	in-depth interviews	physicians (*n* = 49, medical specialists, GPs and elderly care physicians) and members of the general public (*n* = 16)
Snijdewind et al. [79]	2016	To show what developments physicians see in practice and how they perceive the influence of the public debate on the practice of EAS.	in-depth interviews	physicians with experience with complex EAS cases (n = 28)
Cate et al. [12]	2017	To gain a better understanding of the considerations that play a role when physicians decide on a request for EAS	in-depth interviews	GPs (*n* = 33)
Miller et al. [69]	2017	To assess how Dutch regional euthanasia review committees apply EAS due care criteria in cases where the criteria are judged not to have been met and to evaluate how the criteria function to set limits in Dutch EAS practice.	content analysis, qualitative	reports of EAS cases where the due care criteria were not met (*n* = 32)
Quantitative Studies				
van der Wal et al. [57]	1991	To examine the suffering of patients who died by euthanasia or assisted suicide	survey, retrospective, questionnaires	patient cases (*n* = 388) of GPs
van der Wal et al. [53]	1992	To determine whether Dutch family doctors acted prudently with regard to EAS	survey, retrospective, questionnaires	GPs (*n* = 667)
Muller et al. [61]	1996	To obtain insight into the involvement of a patient’s social network in the process leading to the administration of euthanasia and assisted suicide	survey, retrospective, questionnaires	GPs (*n* = 1044), nursing home physicians (*n* = 713)
Verhoef et al. [54]	1997	To describe the incidence of euthanasia and assisted suicide in family practice in the Netherlands, the reasons for its practice, and the characteristics of patients and physicians involved	survey, cross-sectional, questionnaires	GPs (n = 667)
Willems et al. [91]	2000	To compare attitudes and practices concerning the end-of-life decisions between physicians in the United States and in the Netherlands	survey, interviews with vignettes	clinical specialists (internists) from the Netherlands (*n* = 67) and Oregon, USA (*n* = 152)
Swarte et al. [77]	2003	To assess how euthanasia in terminally ill cancer patients affects the grief response of bereaved family and friends	survey, questionnaires	family members and close friends of cancer patients who died by euthanasia (*n* = 189), control group of cancer patients who died a natural death (*n* = 316)
Heide et al. [67]	2003	To investigate the frequency and characteristics of end-of-life decision making in six European countries	survey, questionnaires	attending physicians related to death certificates (*n* = 20,580)
Groenewoud et al. [55]	2004	To describe the practice of psychiatric consultation with regard to explicit requests for euthanasia or physician-assisted suicide in the Netherlands	survey, questionnaires	psychiatrists (*n* = 549)
Jansen et al. [51]	2005	To obtain information about the characteristics of requests for euthanasia and physician-assisted suicide (EAS) and to distinguish between different types of situations that can arise between the request and the physician’s decision	survey, questionnaires	GPs (*n* = 3614)
Rurup et al. [59]	2005	To determine how often EAS requests are made in the absence of severe disease and how such requests are dealt with in medical practice in The Netherlands	survey, interviews	Nursing home physicians (*n* = 77), GPs (*n* = 125), clinical specialists (*n* = 208)
Rurup et al. [70]	2005	To estimate the incidence of (compliance with) advance euthanasia directives of patients suffering from dementia in the Netherlands and to gain knowledge about the experiences of physicians	survey, interviews	Nursing home physicians (n = 77), GPs (*n* = 125), clinical specialists (*n* = 208)
Vrakking et al. [85]	2005	To study the willingness of Dutch physicians to use potentially life-shortening or lethal drugs for severely ill children	survey, interviews	pediatricians (*n* = 63), GPs (n = 125), clinical specialists (n = 208)
Rietjens et al. [94]	2006	To gain insight in thecharacteristics of a good death for the Dutch general population, and to identify whether attitudes towards euthanasia, terminal sedation and using high dosages of morphine are associated with attitudes towards a good death.	survey, questionnaires	members of the general public (*n* = 1388)
Rurup et al. [95]	2006	To investigate the attitude of physicians, nurses and relatives towards medical end-of-life decisions concerning patients with dementia	survey, questionnaires	nursing home physicians (*n* = 107), relatives of patients with dementia (*n* = 136), nurses (*n* = 178)
Georges et al. [66]	2007	To describe the experiences of patients who died by euthanasia or physician-assisted suicide (EAS) in the Netherlands	survey, interviews	relatives of patients who died by EAS (*n* = 87)
Bruchem et al. [83]	2007	To describe the role of district nurses in euthanasia and physician-assisted suicide in home care organizations	survey, questionnaires	district nurses (*n* = 408) employed in homecare organizations
Vrakking et al. [86]	2007	To gain insight into how Dutch pediatricians and other physicians treating children feel about the regulation of physician-assisted dying	survey, interviews	pediatricians (*n* = 63), GPs (n = 125), clinical specialists (n = 208)
De Veer et al. [84]	2008	To investigate the views of nurses on their role with regard to the discussion of end-of-life decisions, as well as their actual role in the decision-making process	survey, questionnaires	Nurses (*n* = 489) involved in palliative care
Hanssen-de Wolf et al. [63]	2008	To investigate which sources physicians use to determine whether three of the due care criteria (unbearable and hopeless suffering, and no realistic alternatives for treatment) are met.	survey, questionnaires	GPs (n = 3614)
Abarshi et al. [52]	2009	To explore the relationship, if any, between EAS requests and cancer types, in relation to the provision of end-of-life care in the Netherlands	Survey, questionnaires	GPs (*n* = 3614)
Rietjens et al. [88]	2009	To evaluate the extent to which general practitioners (GPs), consulted physicians and members of the euthanasia review committees judge the criterion of unbearable and hopeless suffering similarly	survey, questionnaires with vignettes	GPs (*n* = 300), consultants (*n* = 150), members of review committees (n = 27)
Tol et al. [87]	2010	To examine what doctors consider ‘unbearable suffering’ and for what kind of suffering they are willing to grant a euthanasia request.	survey, questionnaires with vignettes	GPs (*n* = 115)
Ruijs et al. [48]	2013	To study symptom prevalence, the relationship between intensity of symptoms and unbearable suffering, the evolvement of symptoms and unbearability over time and quality of unbearable suffering	survey, prospective, interviews	end-of-life cancer patients in primary care (*n* = 64)
Booij et al. [40]	2014	To investigate the presence of thoughts about or wishes for the end of life in patients with Huntington’s disease (HD) or identified gene carriers (further mentioned together as patients)	survey, questionnairs	patient with Huntington’s disease and identified gene carriers (*n* = 242)
Brinkman-Stoppelenburg et al. [90]	2013	To study why euthanasia requests are sometimes judged not to meet the requirements of due care and to find out which characteristics are associated with the SCEN physicians’ judgments.	survey, questionnaires	SCEN physicians (*n* = 415)
Maessen et al. [50]	2014	To determine if quality of care, symptoms of depression, disease characteristics and quality of life of patients with amyotrophic lateral sclerosis (ALS) are related to requests for euthanasia or physician-assisted suicide (EAS) and death by EAS	survey, prospective, questionnaires	ALS patients (*n* = 102)
Ruijs et al. [49]	2014	To study the prevalence of unbearable symptoms and overall unbearable suffering in relationship to explicit requests for EAS in a cohort of end-of-life cancer patients in primary care	survey, prospective, interviews	end-of-life cancer patients in primary care (*n* = 64)
Buiting et al. [58]	2009	To study which arguments Dutch physicians use to substantiate their adherence to the due care criteria and which aspects attract the attention of review committees’	content analysis	physicians’ reports and verdicts of review committees for granted EAS cases (*n* = 158)
Heest et al. [80]	2009	To reveal how often and in what way palliative sedation and euthanasia were discussed in telephone consultations with GP advisors in palliative medicine	content analysis	documentation forms for telephone consultations (n = 415)
Snijdewind et al. [92]	2015	To study outcomes of requests for euthanasia or physician-assisted suicide received by the clinic and factors associated with the granting or rejecting of requests	content analysis	registration files of patients who applied to the End-of-Life Clinic (*n* = 645)
Kim et al. [60]	2016	To describe the characteristics of patients receiving EAS for psychiatric conditions and how the practice is regulated in the Netherlands	content analysis	summaries of psychiatric EAS cases made available by the review committees (*n* = 85)
Mixed-method Studies				
van der Maas et al. [56]	1991	To present the first results of the Dutch nationwide study on euthanasia and other medical decisions concerning the end of life (MDEL).	interviews, questionnaires, prospective survey	Physicians (interviews and prospective survey *n* = 405 and 322, resp. Questionnaires: *n* = 5197)
van den Boom [74]	1995	To study the relationship between AIDS, euthanasia and grief	questionnaires and interviews	Relatives of deceased AIDS patients (*n* = 60)
Haverkate et al. [82]	2000	To determine the prevalence of EAS guidelines in Dutch nursing homes and to analyze the content.	questionnaires and content analysis of guidelines	Nursing home directors (*n* = 313), guidelines (*n* = 183)
Vrakking et al. [72]	2005	To assess the frequency of end-of-life decisions preceding child death and the characteristics of the decision-making process in the Netherlands	death certificate study, questionnaires, interviews	death certificates (*n* = 129), involved physicians (n = 63)
Rurup et al. [43]	2006	To investigate the prevalence of ADs and the factors associated with the formulation of an AD in The Netherlands	questionnaires, interviews	members of the general public age < 60 years (*n* = 1051), > 60 years (*n* = 3107), relatives of patients who died after EAS (*n* = 87)
Van der Heide et al. [62]	2007	To assess the effects of the 2002 Dutch euthanasia law and changes in end-of-life care	death certificate study and questionnaires	Death certificates, attending physicians (*n* = 6860)
Jansen-van der Weide et al. [75]	2009	To investigate the impact of a visit from a consulting physician on patients and relatives during the euthanasia procedures in the Netherlands	surveys and interviews (both questionnaire-based and in-depth)	GPs (n = 3614) and relatives (*n* = 86)
Onwuteaka-Philipsen et al. [93]	2010	To study which patients request euthanasia and which requests actually result in euthanasia in relation with diagnosis, care setting at the end of life, and patient demographics.	death certificate study and questionnaires	Death certificates, attending physicians (n = 6860)
Buiting et al. [76]	2011	To investigate how good SCEN consultations differ from less satisfactory SCEN consultations	questionnaires and qualitative interviews	*questionnaires:* euthanasia consultants (*n* = 594) *Interviews:* euthanasia consultants (n = 14), physicians who had requested an euthanasia consultation (n = 12), relatives (n = 10)
De Boer et al. [71]	2011	To gain insight into how ADs for euthanasia affect resident care in nursing homes	questionnaires and in-depth interviews	*questionnaires:* elderly care physicians (*n* = 434) *Interviews:* elderly care physicians (n = 11) and relatives (n = 8) of deceased dementia patients with euthanasia AD
Van Delden et al. [42]	2011	To investigate knowledge, opinions and experiences regarding euthanasia, palliative sedation and ADs among health care professionals and members of the general public.	questionnaires and in-depth interviews	*questionnaires:* members of the general public (*n* = 1960), physicians (*n* = 793), nurses (*n* = 1243) *Interviews:* physicians (*n* = 49), nurses (n = 18), members of the general public (*n* = 16)
Van der Heide et al. [47]	2012	To examine the practice of medical decisions at the end of life after 10 years of the Euthanasia Act, how the regulations of the Euthanasia Act are applied and which problems occur	questionnaires and in-depth interviews	*questionnaires:* physicians (*n* = 2000) *interviews*: physicians (n = 30, 22/30 GPs), relatives of patients who died of EAS (*n* = 25)
Onwuteaka-Philipsen et al. [7]	2017	To determine whether the Euthanasia Act reaches its aims	survey and in-depth interviews	*questionnaires:* physicians (*n* = 2500), members of the general public (*n* = 2500) *interviews*: psychiatrists (*n* = 17)
Bolt et al. [73]	2017	To explore the situations in which pediatricians found PAD conceivable and to describe the roles of the patient and parents, the patient’s age and their life expectancy	survey and in-depth interviews	pediatricians (*n* = 276 and n = 8)

Abbreviations: *GP* general practitioner, *EAS* euthanasia or assisted suicide, *AD* advance directive, *SCEN* support and consultation euthanasia Netherlands

In the thematic analysis of the included studies, 19 descriptive themes were identified and four overarching analytical themes were developed. The descriptive and analytical themes are displayed in Table 3 and described in detail in the section below. It was found that the concepts family, family members, relatives, social network, friends and others were used interchangeably across the different studies, often without further clarification of the concepts. An attempt to define the patient’s significant others involved in EAS is found under theme 2.1. Recent and detailed quantitative data necessary for a solid definition turned out to be missing, however.

**Table 3:** Analytical and descriptive themes

1. Family-related reasons (not) to make a request for EAS: Fear of suffering as witnessed previously in other family members ▪ Family beliefs and dynamics ▪ Importance of maintaining meaningful bonds ▪ Feeling of being a burden ▪ Quantitative research among physicians: other reasons more important	
2. Roles and responsibilities of family members during EAS decision-making and performance: Social network involved in decision-making and performance ▪ Sounding board for patient and physician ▪ Caregiving, representing, advocating ▪ Negotiating the date of performance ▪ Proxy-decision making: children, patients with dementia	
3. Families’ experiences and grief process after euthanasia and assisted suicide: Ambivalence, exhaustion, difficulty of choosing a date of performance ▪ Varying experiences related to the interaction with physicians ▪ Mainly positive evaluations afterwards ▪ Complicated grief after a complicated process	
4: Family and the ‘good euthanasia death’ according to Dutch physicians: Physicians’ experiences with EAS and family involvement ▪ Taking care of the family as a task ▪ Family support or agreement as additional criterion for EAS ▪ Reluctance to consider social indications for EAS ▪ The general public’s opinion on family’s involvement in EAS	

### Theme 1: family-related reasons (not) to make a request for euthanasia or assisted suicide

Considerations about family members and the broader social network were frequently found to play a role in why people made a request for EAS, postponed or withdrew it.

#### Fear of suffering as witnessed previously in other family members

In all three ethnographic studies and several other qualitative studies, patients are described whose request for euthanasia or assisted suicide is motivated by a fear of the suffering, dependency, uncertainties or strain on caregivers they had witnessed previously surrounding the deathbed of partners, parents or siblings [9, 34–38]. For instance, patients with cancer and AIDS expressed the wish to prevent the suffering they had witnessed at the deathbed of parents or partners by choosing EAS [9, 35]. Family members of patients with cancer recounted similar stories when asked about the patient’s reasons to request euthanasia [34, 38]. In-depth interviews with patients with dementia showed how instructions regarding euthanasia were made in advance following experiences with family members, most often parents, or others they had cared for who were afflicted with dementia [39].

In an interview study, patients with Huntington disease spoke “frequently and spontaneously” about experiences with ill parents or other blood relatives in relation to their wish to have control over the end of their life [37]. In a parallel questionnaire-based study, any thoughts about end-of-life wishes, including EAS, were significantly related to family experiences with the disease, and not to clinical or demographic variables [40]. Similarly, members of the general public referred to situations of decay, pain and humiliation they had seen in family and friends who were afflicted with metastasized cancer or dementia, to explain their positive opinions regarding euthanasia and advance euthanasia directives [41, 42]. Furthermore, ‘experience with EAS in the environment’ was one of the factors significantly related to having an advance euthanasia directive, next to other factors such as being a women, being non-religious, a high education level, the death of a marital partner, inadequate personal care support and several illness-related factors [43].

#### Family beliefs and dynamics

In several studies, patients and their partner, children or siblings describe how the wish for EAS was part of a personal philosophy of life, developed together long before becoming ill [9, 38, 44]. In one family, both parents and children were members of the right-to-die association [9]. Some patients spoke explicitly about the goal to have a well-organized farewell and aftermath for the sake of their family members [9, 38]. In other instances, family members and physicians related the request for EAS to someone’s position in the family and his/her character [9, 38, 45]. A single case was mentioned of an EAS request relating to a grave family secret [38].

#### Importance of maintaining meaningful bonds

In qualitative studies on physicians’ and nurses’ experiences with EAS, the healthcare professionals describe how patients’ growing inability to connect meaningfully to significant others can motivate the request for EAS. They describe patients who wanted EAS as soon as they were no longer able to recognize children or partners, or could no longer enjoy their company due to illness-related symptoms [9, 42, 44, 46]. In contrast, there were cases where patients enjoyed positive experiences with for example grandchildren, in conjunction with postponing or being ambivalent regarding their own request for EAS [9, 34, 37]. For some patients, concerns about family members not being able to bear EAS or not being ready for it were reasons to postpone a request for EAS or not to disclose it to their family members [9, 36, 38]. Euthanasia-requests relating to loss of a partner [42] and loneliness [34] were described as well.

#### Feeling of being a burden

In several qualitative studies, nurses and physicians recount stories of euthanasia-requests related to dependency on the care of children or partners and the associated burden on them [9, 35, 47]. Financial burdens were only mentioned twice in relation to EAS in all included studies, by a patient without insurance who was worried about the financial consequences for his wife [38] and a young AIDS patient who said to prefer euthanasia above suicide because of his life-insurance [9].

##### Several themes combined

Norwood’s ethnography, conducted in a primary care setting, offers an illustrative example of the themes mentioned above [35]. She describes an 82-year-old cancer patient who requested euthanasia because of unbearable suffering, meaninglessness, and the wish not to be a burden to her daughter. She cancelled it out of consideration for her daughter who was not ready for it yet, regretted the cancellation, but later on enjoyed the company of her granddaughter again. In her comprehensive in-depth interview study on unbearable suffering in patients with an explicit request for euthanasia, Dees et al. describe how medical, psycho-emotional, socio-environmental and existential elements, as well as the person’s biography and character, together constitute the suffering that led to a request for EAS [45]. While the socio-environmental elements found in Dees’s study largely resemble the above-mentioned family-related reasons, psycho-emotional and existential elements such as hopelessness seemed to contribute more to unbearable suffering. In a prospective survey involving end-stage cancer patients in primary care, fear of future suffering and the feeling of being a burden, together with loss of autonomy and physical suffering, were reported more frequently in patients who suffered unbearably [48]. However, neither this study [49] nor a prospective survey among ALS patients [50] found a significant difference in the prevalence of those symptoms between patients with and without an explicit request for EAS.

#### Quantitative research among physicians: other reasons more important

Whereas qualitative studies turned up a variety of family-related reasons for EAS, this theme was less prominent and emerged differently in quantitative studies in which physicians were asked about their patient’s main reasons to explicitly request EAS. Both in studies with GPs [51–54] and with (consulting) psychiatrists [7, 55] who had been involved in the care for patients with an explicit request for EAS, ‘feeling of being a burden’ was mentioned as a reason, yet it was among the less important and less frequently cited reasons. Hopelessness and unbearable suffering [51–55], loss of dignity [51, 52, 56], pain [56, 57], tiredness/weakness [51, 52, 57], and dependency [56, 57] were mentioned by physicians as the most important reasons. This pertained both to patients with an underlying physical disorder and with a mental disorder. In one study involving psychiatrists who had cared for psychiatric patients with an explicit request for EAS, the most important reasons cited were depression, major problems in various aspects of life and loss of control [7]. A study on the content of physicians’ reports about patients who received EAS (underlying illness not specified) showed how, in the majority of cases, the suffering was explained in terms of physical symptoms, followed by loss of function, dependency and deterioration [58]. ‘Being a burden’ and loneliness were again mentioned, but relatively rarely. In the study by Jansen et al., GPs mentioned ‘not wanting to be a burden’ more frequently as a reason for an explicit request in patients whose request had been refused [51]. Additionally, in a study on requests for EAS in the absence of a severe disease, physicians described the most important reasons in terms of being weary of life and physical decline, while the patients’ actual problems were mostly characterized as of social origin [59]. Finally, in a recent content analysis of summaries of psychiatric EAS cases provided by the review committees, loneliness and social isolation were mentioned as an important element in 56% of cases, in addition to the psychiatric morbidity [60].

### Theme 2: roles and responsibilities of family members during EAS decision-making and performance

#### Social network involved in decision-making and performance

Both qualitative and quantitative studies show that once a patient has made a request for EAS to a physician, a process of deliberation, decision-making and finally performance starts in which family members seem to be thoroughly involved [7, 9, 11, 34–36, 38, 42, 44, 61–64]. Partners, children and siblings were most frequently mentioned as the patient’s significant others who were involved in the process of decision-making [9, 34–36, 38, 44, 61, 64, 65]. Some studies describe how friends [35, 38], neighbors [11], or nurses [34, 35, 38] created a supportive social network during EAS decision-making and performance in cases where family was absent. In two qualitative studies, patients with psychiatric diseases are mentioned who explicitly refused any contact with family members about their request for EAS [7, 64]. Georges’s survey interviews found that most patients had discussed their end-of-life whishes with their family members, mostly partners and children, before explicitly requesting EAS [66]. Muller’s questionnaire-based study from 1996 shows how, in approximately 90% of cases of EAS, GPs and nursing home physicians (NHPs) spoke with the social network, mostly partners and children, about the request, the intention to grant it and how it would be performed [61]. NHPs spoke slightly more often with ‘other family’ and ‘others’, though both categories were not specified any further. Recent quantitative studies have found slightly lower percentages of physicians who discuss the decision to perform EAS with family members [7, 62], while Dutch physicians are still found to speak most frequently about end-of-life decisions with family members, compared to their counterparts in other European countries [67]. In qualitative studies, the actual performance of EAS is described as happening in the presence of partners, parents, siblings and sometimes friends at the bedside [9, 34–36, 38, 44, 64, 68]. According to Muller’s study from 1996, partners and children were most often present at the bedside in the homecare setting, while for one-third of patients receiving EAS in nursing homes, no-one from the social network was present [61]. Verhoef et al. described in 1997 how 96% of EAS-deaths took place at home, of which 99% in the presence of others [54]. Another study described a single case of a patient who did not want anyone to be present at the bedside during the administration of EAS [42].

#### Sounding board for patient and physician

Ethnographies and other interview studies with patients and family members describe how patients asked their family members to consent to EAS [37, 38], how euthanasia-declarations were drawn, signed and discussed collectively [9, 34, 35, 37, 38, 65], and how alternatives and procedures for EAS were discussed among patient, family and physician [9, 34–36, 44, 64]. In qualitative interviews with and observations of physicians, it is described how physicians test their impression regarding the voluntariness of the request and the unbearableness of the symptoms in repeated discussions with family members [35, 38]. Likewise, Hanssen’s quantitative study showed that GPs had conversations with the patient in 95% of cases and with family members in 71% of cases to come to a judgment about unbearable suffering [63].

#### Caregiving, representing, advocating

Case studies and Norwood’s ethnography describe how husbands, wives, siblings, parents and children and also a neighbor acted as informal caregivers during EAS decision-making in general practice, with or without help of professional caregivers [35, 36, 44, 65, 68]. Only Dees et al. lists caregiving responsibilities among the characteristics of family members who participated in their study on EAS decision-making [64]. The care they provided ranged from zero (a nephew) to twenty-four hours a day (husbands and wives).

In addition, family members acted as patients’ advocates as well as family representatives in conversations with physicians, as other ethnographic studies and the in-depth interviews of Ciesielski-Carlucci et al. found. For instance, partners, children or siblings are described who informed the physician about a worsening of symptoms [9, 34, 38], signaled the right moment for the performance of euthanasia [38, 44] or initiated the discussion about EAS on behalf of competent patients who suffered from a progression of the underlying physical disorder [9]. The describes the case of a sister who initiated the conversation about a euthanasia request with the physician, on behalf of her brother whose cognitive abilities were fluctuating due to brain metastases [34]. In an earlier stage of illness, the patient and his sister had already talked extensively about the possible scenarios of illness progression and the request for euthanasia, and the sister had been appointed as his representative. In a study of EAS cases that the regional review committees disapproved of, single cases were described of family members who helped the physician administer lethal medication or who organized an appointment for a family member afflicted with dementia at the specialized End-of-Life clinic [69].

#### Negotiating the date of performance

Case studies and ethnographies describe how EAS was planned together with family members, taking into account time for leave-taking, family members traveling from abroad, family holidays, birthdays or responsibilities at work [9, 36, 68]. This is echoed in interviews with family members and physicians, who explained that as long as the physical suffering was not too acute or severe, these kinds of family-related considerations were taken very seriously [47]. In contrast, Dees et al. describe how most family members in their study preferred to stay out of the planning of EAS, and that the patient and physician choose a date, depending on many other factors like fears about symptom progression, loss of competency and psychological suffering [64].

#### Proxy-decision making: children, patients with dementia

Proxy-decision making came explicitly to the fore in studies about euthanasia for patients with dementia and euthanasia for children. A retrospective interview study with nursing home physicians (NHPs) from 2005 described how, in the majority of cases, NHPs discussed advance euthanasia directives by patients with dementia with the patient’s family members or representatives [70]. According to the NHPs in this study, family members wanted to discuss the advance euthanasia directives most often with the purpose of discussing end-of-life policies in general. From a mixed-method study and a qualitative interview study about advance euthanasia directives drawn up by patients with dementia, it emerged that family members were sometimes involved in writing, discussing or interpreting the advance directives [39, 71]. However, the drafting of an advance euthanasia directive was most often initiated by patients themselves [71]. Some patients expected their families to act upon the euthanasia advanced directive at the right moment, even if that moment was not clearly specified [39]. Two studies found that advance euthanasia directives were rarely carried out for various reasons, such as nursing home policies [70, 71], doubts about the presence of unbearable suffering or the applicability of the advance directive in that situation [71], and opinions of NHPs about the acceptability of euthanasia for patients with dementia or the validity of a request based on an advance directives [70]. Quantitative studies about end-of-life decisions for children indicate that 25 to 50% of pediatricians at some time receive an explicit request for physician assisted dying for children between the age of 1 and 17, and that in the majority of cases the request comes from parents [72, 73]. The actual performance of physician assisted dying in children was rare according to a study of death certificates (2.7% of deaths in children), but in the few cases that were found, it was mostly at the explicit request of the parents (2.0 out of 2.7%) [72].

### Theme 3: families’ experiences and grief process after euthanasia and assisted suicide

#### Ambivalence, exhaustion, difficulty of choosing a date of performance

Several qualitative studies describe how family members struggle with conflicting feelings during euthanasia decision-making. While they wish for the patient’s suffering to end, and regardless of personal views on EAS, they often considered EAS to be too early, or too definitive [9, 34, 35, 44, 74]. Furthermore, several studies describe family members who had been aware of their own exhaustion due to caregiver responsibilities during euthanasia decision-making [9, 35, 38, 44]. For some it had been a reason to stay out of euthanasia decision-making, or to doubt their role in it [38, 44]. For others, euthanasia was seen as a possibility to organize care and to have a fixed end point of care responsibilities [35]. In various other studies, healthcare professionals -- both physicians and others -- describe exhausted family members, emotionally burdened by the course of events at the deathbed [9, 34, 38, 73]. According to healthcare professionals, families’ exhaustion was sometimes projected onto the patient [9, 34] and an appeal for EAS could be just a call for support and clarity [34]. Meanwhile, planning of EAS could cause relief in exhausted family members [9]. However, the activity of collectively picking a date was often described as very difficult and even as overwhelming or absurd [9, 35, 38, 64].

#### Varying experiences related to the interaction with physicians

Qualitative studies conducted in primary care describe how discussions about euthanasia were a positive experience for family members. The discussion could foster mutual bonds between patient, family and physician, if there was clear communication and respect for all involved [35, 64]. In contrast, other studies mention how family members had great difficulties with the indecisiveness or conflicting messages of physicians about a request for EAS, and how they could feel powerless and not taken seriously as a patient’s representative [34, 47, 73]. Family members were also found to struggle with acting upon a euthanasia advanced directive, which could cause disagreement about whether the state of unbearable suffering had been reached and about who should make the final decision to act upon it [39, 71]. Lastly, a visit by a consulting physician could provoke different experiences as well, ranging from a positive experience because the consultation was seen as a safeguard in the procedure, to negative experiences relating for instance to a negative judgment about the request for EAS [75, 76].

#### Mainly positive evaluations afterwards

After euthanasia has been performed, positive experiences seem to prevail in bereaved family members, according to both the findings of qualitative and a limited number of quantitative studies [7, 9, 34, 36, 38, 44, 47, 64, 66]. Despite the feelings of grief and loss, many family members mentioned that they felt relieved that the suffering had ended, that the patient’s wish had been fulfilled, and that it had been a peaceful deathbed surrounded by loved ones. In one study, a bereaved daughter and son-in-law directly related their positive experiences with the euthanasia-death of their mother to preferences for their own death in the future [38]. In line with these positive experiences, Swarte et al. found statistically significant less traumatic grief in family and friends after EAS compared to a ‘normal’ death in a cross-sectional study performed in a tertiary oncology center [77]. In this study, cancer patients who died through euthanasia more often had ‘others’ as their social network, such as cousins and friends, than cancer patients who died a natural death, which more often involved partners and first-degree blood relatives. In bereaved partners, family and friends of AIDS patients, no significant association was found between the occurrence of depression after a normal versus a euthanasia-death [74].

#### Complicated grief after a complicated process

Nevertheless, complicated grief and negative experiences after euthanasia were described as well, relating to a hampered process preceding EAS [38, 47, 68, 74] or to secrecy among close family members about EAS as cause of death [35, 38]. In his 1995 mixed-method study with AIDS patients, Boom found how complicated grief can be due to the responsibility of deciding about the date for EAS, the speed of dying after the lethal injection (extremely fast or prolonged), and the responsibility for administering lethal medication. The latter aspect was mentioned in a case study as well [68]. In addition, in two studies from the nineties, single cases are described of family members who developed psychopathology, or who thought of or actually committed suicide using lethal drugs that were at a patient’s disposal [38, 74]. Furthermore, three qualitative studies investigating family members’ experience after EAS mention how some family members refused to participate in the study due to the sensitivity of the topic or the emotional burden after euthanasia [66, 71, 75].

### Theme 4: family and the ‘good euthanasia death’ according to Dutch physicians

#### Physicians’ experiences with EAS and family involvement

Dutch physicians’ personal experiences with EAS seem to be profoundly influenced by the involvement of family members in both positive and negative ways. This applies especially for GPs. Although EAS was described as heavy and burdening regardless of one’s personal views [38, 47], GPs found comfort and had positive experiences thanks to the support and expressions of gratefulness of bereaved family members [35, 36, 38, 44, 78]. Even the experience of ‘becoming part of the family’ was mentioned in a few studies [35, 38, 47]. At the same time, complexities and negative experiences with EAS for GPs and other physicians were found to relate to the involvement of family members as well. The pressure that family members exerted on the physician, disagreements about the process or the suffering, opposition, lack of open conversation, different expectations and the idea that euthanasia was an enforceable right were described as elements of a complex or negative EAS process [9, 11, 35, 38, 42, 47, 64, 79]. Some consulting physicians had similar negative experiences relating to family pressure [65, 76], and family-related complexities could be a reason for consultation with other GP advisors in palliative care [80]. However, the 2011 survey by Van Delden et al. found that only 13% of participating physicians had negative experiences with pressure exerted by family members during EAS decision-making. Almost 90% of the physicians experienced respect for their position and were able to come to an agreement about the final decision [42].

#### Taking care of the family as a task

From qualitative studies conducted among GPs, it emerged that many, although not all of them, considered it their task to take care of both patient and family members during euthanasia decision-making [7, 9, 11, 35, 38, 44, 47, 64, 81]. Specific elements of taking care were described, namely establishing good relationships with all involved [11, 35, 38, 64, 81], informing and preparing family members for EAS [35, 38, 47, 64], facilitating contact with estranged family members [35, 38] and taking into account the family members’ future grief process [9, 35, 38]. Some GPs describe how ‘euthanasia-talk’ could have a therapeutic effect for all involved, reducing anxiety and fostering open conversation about death [35, 38]. Similar themes were mentioned in recent interviews with psychiatrists who have experience with EAS [7]. On the other hand, some GPs and other physicians were described who only see taking care of their individual patient as their task [9, 11, 38]. One study on EAS guidelines in nursing homes showed how 75% of these guidelines mention that consultation with or the informing of family members is advised [82]. Studies involving nurses showed that although a minority of nurses is actually involved in EAS decision-making, they often consider it their task to counsel both patient and family members and to provide aftercare for family members [34, 83, 84].

#### Family support or agreement as additional criterion for EAS

Both earlier and recent qualitative studies among GPs and psychiatrists describe how these physicians considered to forego euthanasia in cases where the family could not cope, where family was absent or if there was a family conflict, despite knowing that these considerations were not related to any legal criterion [7, 11, 12, 35]. Furthermore, in a study investigating physicians’ opinions about EAS for children, GPs and medical specialists less often agreed with the idea of granting EAS for a child without parental consent than pediatricians [85]. Diverging opinions among pediatricians on the extent to which parents should be involved in EAS decision-making for children were found in other studies as well [73, 86].

#### Reluctance to consider social indications for EAS

Besides family agreement as additional criterion, a reluctance to consider EAS on social indications was found as a theme in both quantitative and qualitative studies, especially in studies with GPs. A vignette-based study found a substantial variation in opinions among GPs whether the suffering in vignettes marked as ‘being a burden’, ‘dependency’ and ‘fear of future suffering’ could be judged as unbearable suffering, with a 88% correspondence with not granting an EAS request [87]. In addition, GPs were significantly less likely to judge fictional cases marked as ‘being a burden’, ‘dependency’ and ‘future decay’ as unbearable suffering compared to consulting physicians and members of review committees, while they mostly agreed on the presence of unbearable suffering in a case marked by acute pain and progressive symptoms due to a physical disorder [88]. Another survey showed how only a minority of GPs would consider granting an EAS request without an underlying somatic or psychiatric disorder [89]. Similarly, qualitative studies described a GP who organized additional care to assure that a request did not originate from the feeling of being a burden [38], as well as GPs and other physicians who found it difficult to empathize with EAS requests based on dependency, loneliness and existential aspects [12, 79]. Snijdewind et al. describe how several, but not all physicians, doubted whether physicians should have a role in such cases and whether these were medical or societal problems [79]. Furthermore, ‘being a burden’ as an important part of unbearable suffering was related to a higher likelihood that consulting physicians took a negative view of the legal criteria [90] and to less willingness on the part of Dutch internists to perform EAS, compared to their counterparts in the US [91]. In addition, a recent study showed how the rejection of patients in the specialized End-of-Life clinic was significantly related to being single or without children, while granted requests were independently associated with having more than one child [92]. A study from 2010 based on death certificates found no association between marital status and refusal of EAS requests [93].

#### The general public’s opinion on family’s involvement in EAS

In contrast to physicians’ reluctance to consider requests for EAS on social indications, a questionnaire-based study about notions of ‘good ways to die’ among the general public showed how an acceptance of euthanasia was significantly related to reasons like avoiding being a burden on others and dependency, in addition to remaining in control and having a painless death [94]. An earlier questionnaire-based study described more permissive attitudes among relatives compared to physicians regarding (proxy-decision making for) EAS for patients with dementia [95]. At the same time, opinions were divided about whether it was important for family members to be involved in EAS decision-making. An earlier mixed-method study among the general public found instances of fear about bad intentions of family members and concerns about their influence on EAS decision-making [42].

## Discussion

The aim of this review was to explore how family members are involved in the Dutch practice of EAS according to existing empirical research, and to map out themes relevant for further research and discussion. The results indicate that a request for EAS can originate from family-related considerations, that family members seem to fulfill demanding roles and responsibilities in EAS decision-making with varying experiences, and that Dutch physicians - especially GPs - seem to show sincere consideration for family members and the broader social context when deciding on a request for EAS. The results of this review offer a new perspective on EAS decision-making in the Netherlands, which is typically framed in the patient-physician dyad. However, a triad model in which family members also have a position seems more appropriate to describe what goes on in clinical practice, as suggested previously by Snijdewind et al. as well [11]. Adopting such a patient-physician-family triad for EAS decision-making brings empirical and ethical questions to attention that have not been sufficiently addressed so far.

### Methodological considerations

It is important to acknowledge the limitations of the explorative approach and qualitative method of synthesis used in this review. Due to the heterogeneity of study aims, methods and participants in the included studies, the generalizability of the results is limited. Also, it is particularly challenging to synthesize qualitative studies since the context may partly be lost in the process, while the context is essential for a correct interpretation of the results. Besides, the context of several of the included studies may no longer be comparable to the present-day practice of EAS, which is described in more detail below. Nevertheless, this review raises themes and identifies problems that can serve as a starting point for further empirical and ethical inquiry.

One may criticize the inclusion of studies in which family members themselves weren’t study participants or in which their experiences and roles were not the main subject of inquiry. However, the number of studies with family members as participants was found to be limited (14 out of 66 studies), which may itself be considered a significant result. Additionally, the literature search yielded studies that mentioned patients’ and physicians’ perspectives on and experiences with family members or the broader social context. Since the objective of this review was to explore this underdeveloped field, a broad interpretation of “involvement of family members” was chosen, in order to highlight a wide range of themes that might be relevant for further inquiry and debate.

### Implications for empirical research

#### Question 1: the patient’s family or significant others…who are they, really?

First and foremost, the question is who the patient’s significant others are who could be involved in the present-day practice of EAS in the Netherlands. There is no standardized registration of the people in the social networks of patients who request and receive EAS. For instance, physicians who perform EAS are not required to report this kind of information to the regional euthanasia review committees. In this review, partners and children feature as the most closely involved significant others. However, the composition of families and social networks seems to be changing in the Netherlands, as is the amount of informal care that the state expects the social networks to provide. Therefore, there is a need for up-to-date quantitative data about the social networks surrounding patients who request and receive EAS in the Netherlands, including information about their caregiver responsibilities and burden. This point has been raised by others as well [1, 91]. The physical, emotional and financial burden of family carers in Dutch general practice and the correlation between caregivers’ burden and patients’ symptoms has been studied before, but not in relation to requests for EAS [96–98]. Quantitative information about professional caregivers other than physicians who are involved in the care for patients and their families, especially in general practice, would be interesting as well. An important theoretical question underlying this is whether the underrepresentation of the social network as study-object or study participants in empirical research is related to their absence in the Dutch euthanasia law.

#### Question 2: how important are family-related reasons for EAS?

This review raises the question how important family-related reasons are for Dutch patients who consider requesting euthanasia or assisted suicide, and how this should be studied. A relationship between the wish to hasten death and witnessing the suffering of others [99] or the feeling of being a burden [100] has been reported before. A recent review on the experiences of patients with a wish to hasten death also described how social-relational factors could be a source of suffering and a reason for expressing a wish to hasten death [101]. However, these social factors were among a range of sources of suffering like physical, psychological and existential factors, and among other reasons for and meanings of the wish to hasten death. Still, the results of this review raise some questions. The presence of family-related reasons in qualitative studies contrasts with the relative absence of them in quantitative studies on reasons for EAS, as recounted by physicians. This is perhaps just a consequence of methodological factors such as study type, included patients/cases, or the stage of decision-making. It may also reflect the physicians’ reluctance to perform EAS on social indication. On the other hand, we may wonder whether physicians recognize family-related reasons or social-relational origins of suffering, and whether patients feel free to speak about it when they have an explicit request for EAS. New qualitative studies with ethnographic or narrative approaches could shed light on how requests for EAS develop over time and in mutual interaction between patients, their significant others and professional caregivers. This approach might also be better suited to identify any cultural, political and existential views that may lie behind requests for EAS [102].

#### Question 3: what about the roles, responsibilities, experiences and grief of the family?

Further in-depth inquiry into the responsibilities and experiences of family members during and after EAS decision-making is necessary. Detailed descriptions of family members’ responsibilities and experiences were mainly found in ethnographies and in-depth interviews from 2009 and before. Some of them were even conducted before the euthanasia law was enacted [9, 34, 38] or were conducted in a hospital [9, 34], while the majority of EAS cases are currently carried out in general practice. Physicians’ practices and the public awareness of EAS have probably changed over the last 20 years. Hence, the interaction between patients, families and physicians during EAS decision-making and related experiences and needs may have changed as well. Furthermore, the quantitative studies on bereavement after EAS included in this review were conducted more than 15 years ago in a hospital setting [74, 77], which is again no longer representative for the majority of cases of EAS nowadays. Empirical studies from the US [103] and Switzerland [104] and grey literature from the Netherlands [105] highlight family members’ ambivalent feelings and demanding tasks when involved in assisted dying, and contrasting findings on grief after assisted dying have been reported [106, 107]. Although these findings resemble the results of this review, further research specific for the Dutch context is needed, such as the work performed by Dees et al. [64], because of differences in legislative frameworks and the pivotal role of general practitioners in the Dutch practice of assisted dying. Future studies could also examine differences in family members’ responsibilities and experiences in cases of euthanasia, compared to physician assisted suicide (PAS) in the Dutch setting. PAS is much rarer than euthanasia in the Netherlands, and interestingly, patients receiving PAS seem affected by psychosocial suffering more frequently than patients receiving euthanasia [108]. In addition, the implications of the use of advance euthanasia directives for both patients, family members and physicians should be explored further. While advance euthanasia directives are receiving steadily more attention in the Dutch public discourse about euthanasia for both patients with somatic as well as patients with dementia, their use in practice seems to be highly problematic [109].

#### Question 4: what about the ‘good euthanasia death’ according to physicians and others?

Lastly, further research is needed to assess the generalizability of results on physicians’ ideas about the ‘good euthanasia death’ and the consequences thereof for clinical practice. Physicians’ varying ideas about the ‘good euthanasia death’ and any additional criteria they may apply might conflict with the wishes of individual patients and the need for clarity about the procedure for both patients and family members. Since GPs are the physicians who currently perform most of the cases of EAS in the Netherlands, it would be valuable to better understand how GPs see their professional role with regard to EAS, what patients’ and families’ expect of their GP, and whether and how these expectations might diverge. For instance, Snijdewind et al. have already described physicians who wonder whether they are seen as “involved caregivers” or “mere performers of EAS” [79].

### Implications for ethical inquiry

Normative conclusions about the involvement of family members in the Dutch practice of EAS would require a thorough examination of ethical arguments on family involvement in medical decision- making and physician assisted dying first, as well as further empirical research as described in the section above. Several scholars have emphasized the moral relevance of family members in medical decision-making, based on the existence of important shared values and interests and the profound influence of family relationships and dynamics on autonomous decision-making and people’s identity [17–19, 24, 110–112]. One of the major ethical issues is whether the interests of individual patients should always prevail, or that the interests and needs of family members should have equal weight or should at least be acknowledged, especially in end-of-life settings [24, 26, 113]. In addition, others have warned about the importance of family dynamics and interpersonal influences in assisted suicide, whether it is medically assisted or not, and how that could infringe on the patient’s responsibility and choice [114]. A close examination of those arguments and how they relate to the Dutch practice of EAS goes beyond the scope of this paper. Still, important suggestions for further ethical inquiry can already be given based on the results of this review.

The results of this review point to a tangle of needs, experiences and responsibilities of patients, their families and physicians in the practice of EAS. These findings already challenge the current Dutch ethical-legal framework of EAS which is based on autonomy, i.e. the voluntary request of the patient, and compassion, i.e. the relief of suffering by the physician [115]. Situating autonomy and the relief of suffering in the patient-physician-family triad, instead of the patient-physician dyad, draws the attention to specific ethical questions.

One of these questions is how a voluntary request for EAS should be both enabled and safeguarded when family members are closely involved in the process of EAS decision-making. The concept of relational autonomy could help examine the different links between relationality and autonomous choice for EAS [11, 18, 19, 111]. Reciprocal and collaborative aspects of autonomy might come into play in EAS, due to the possibility of choice and planning that is typical of assisted dying in contrast to a natural death [22]. However, the normative consequences of a relational concept of autonomy for the practice of EAS should still be examined and discussed [11, 116]. Further, the use of advance euthanasia directives has already been identified as an important ethical issue [109, 117], but the pivotal role and interests of family members may be overlooked if the focus is merely on the question whether an advance euthanasia directive is a sufficient substitute for a voluntary request or not.

Another question is not only what kind, but also *whose* suffering may count in an EAS decision-making process. Dutch physicians have traditionally focused on the patient’s physical suffering as the most important ground for EAS [46], a pattern that was found in this review as well. However, this review has also uncovered family-related reasons for EAS. These family-related reasons could be redefined as suffering because of (experiences with/lack of) significant others, but also as efforts to prevent the suffering of others or to create meaning around the deathbed for all parties involved. It has been argued before that suffering is caused by much more than just physical symptoms [118], and that choosing EAS for family-related reasons could attribute meaning to death and could be fully compatible with autonomous decision-making [119, 120]. Given the divergent opinions among physicians and between physicians and the public about the acceptability of family-related or social reasons for EAS, the concept of suffering and its interpersonal and existential dimensions require further critical examination.

Finally, the patient-physician-family triad in the Dutch practice of EAS draws attention to the “social fabric” that lies behind clinical practice and to questions of professional responsibilities, justice and solidarity [17, 121]. Policy changes that affect the functioning of social networks or the resources of informal caregivers may influence the practice of EAS as well. In addition, the growing focus on autonomous choice in the public debate about EAS in the Netherlands seems to affect expectations regarding physicians’ professional duties [122]. Especially GPs have traditionally focused on the interests of both patients and their family in euthanasia decision-making [121], as found in this review as well. The ethical desirability of this role may be questioned: it can be seen as a way to protect the well-being of both patients and family members, but it could also be seen as an inappropriate use of medical power and as an infringement of individual autonomy [115]. Nevertheless, with the patient-physician-family triad in mind, it seems important to carefully assess what the effect on both patients and their families may be, if the responsibilities of physicians in the Dutch practice of EAS were to change. To conclude: it is still far from clear what a ‘good euthanasia-death’ in the Netherlands should look like, for who and according to who.

## Conclusions

This systematic mixed studies review shows how family members seem to be thoroughly involved at different levels of the Dutch practice of euthanasia and physician assisted suicide. The results reveal how considerations about family members and the social context appear to carry much weight for both patients and physicians when considering a request for EAS. The review also shows how the active participation of family members in EAS decision-making can cause ambivalent feelings and experiences. The results provide a new perspective on the Dutch practice of euthanasia and assisted suicide and challenge the underlying ethical-legal framework, which is based on the patient-physician dyad and the related concepts of autonomy and relief of suffering. Further empirical and ethical inquiry, as well as professional and public debate about the interpretation of the Dutch euthanasia law is needed. Although this review focused on the practice of physician assisted dying in the Netherlands, lessons can be learned for other countries where legislation on physician assisted dying is being considered or has already been implemented.

## Abbreviations

EAS: Euthanasia and physician assisted suicide
GP: General practitioner
DAS: Demedicalized assisted suicide
NHP: Nursing home physician
PAD: Physician assisted dying
PAS: Physician assisted suicide
